# Protective effects of saffron extract and crocin supplementation on fatty liver tissue of high-fat diet-induced obese rats

**DOI:** 10.1186/s12906-016-1381-9

**Published:** 2016-10-22

**Authors:** Maryam Mashmoul, Azrina Azlan, Norhafizah Mohtarrudin, Barakatun Nisak Mohd Yusof, Huzwah Khaza’ai, Hock Eng Khoo, Mehdi Farzadnia, Mohammad Taher Boroushaki

**Affiliations:** 1 Department of Nutrition and Dietetics, Faculty of Medicine and Health Sciences, Universiti Putra Malaysia, 43400 UPM Serdang, Selangor Malaysia; 2 Laboratory of Halal Science Research, Halal Products Research Institute, Universiti Putra Malaysia, 43400 UPM Serdang, Selangor Malaysia; 3 Research Centre of Excellence for Nutrition and Non-Communicable Diseases, Faculty of Medicine and Health Sciences, Universiti Putra Malaysia, 43400 UPM Serdang, Selangor Malaysia; 4 Department of Pathology, Faculty of Medicine and Health Sciences, Universiti Putra Malaysia, 43400 UPM Serdang, Selangor Malaysia; 5 Department of Biomedical Sciences, Faculty of Medicine and Health Sciences, Universiti Putra Malaysia, 43400 UPM Serdang, Selangor Malaysia; 6 Cancer Molecular Pathology Research Center, Imam Reza Hospital, Faculty of Medicine, Mashhad University of Medical Sciences, Mashhad, Iran; 7 Pharmacological Research Center of Medicinal Plants, Faculty of Medicine, Mashhad University of Medical Sciences, Mashhad, Iran

**Keywords:** Saffron extract, Crocin, Fatty liver, Histopathology, Obesity, NAFLD, High-fat diet

## Abstract

**Background:**

Saffron is the dried stigma of *Crocus sativus* L. flower which commonly used as a natural remedy to enhance health and even fights disease in the Middle-East and Southeast Asian countries.

**Methods:**

This study was aimed to investigate protective effect of saffron extract and crocin in fatty liver tissue of high-fat diet induced obese rats. A total of 36 healthy male ﻿Sprague Dawley ﻿rats were divided into six groups. Two groups served as controls, a normal diet (ND) and a high-fat diet (HFD). The other four groups were each supplemented with saffron extract and crocin at concentrations of 40 and 80 mg/kg body weight/day for 8 weeks. All groups except ND were fed with HFD until end of the study. At baseline, blood sample was collected for determination of levels of hepatic marker enzymes, including aspartate aminotransferase, alanine aminotransferase, alkaline phosphatise and albumin. Liver sample was collected, weighed and stained with haematoxylin and eosin for further histopathological examination.

**Results:**

Saffron extract and crocin at concentrations of 40 and 80 mg/kg had dose-dependently alleviated levels of liver enzymes and histopathological changes in diet-induced obese rat model compared to control (HFD group).

**Conclusion:**

This study suggested that saffron extract and crocin supplements have hepatoprotective effect against non-alcoholic fatty liver disease and HFD-induced liver damage.

## Background

Overweight and obesity are major risk factors for medical health problems, such as type 2 diabetes mellitus (T2DM), coronary heart disease (CHD), sleep apnea, cancer and liver disease. Nonalcoholic fatty liver disease (NAFLD) is one of the liver diseases that commonly affect overweight and obese individuals. NAFLD is characterised by abnormal retention of triacylglycerols within liver cell (i.e., hepatocellular steatosis) and the condition can be advanced into more severe liver diseases, such as non-alcoholic steatohepatitis, liver fibrosis, cirrhosis, and not often, liver carcinoma [1]. NAFLD becomes a critical public health issue given its high incidence, likely progression to chronic liver disease, and link with severe cardiometabolic disorders including T2DM and CHD [2]. Noteworthy studies have been engaged in understanding the pathogenesis of NAFLD and designing therapeutic approaches.

Although there is no proven therapy for NAFLD, weight loss and monitoring of the possibly related diseases, such as diabetes mellitus and hyperlipidaemia, are suggested. Two human studies revealed that a moderate, persistent and steady weight loss may lead to an improvement of liver biochemical and histopathological profiles [3, 4]. Since hypertriglyceridaemia and insulin resistance are connected with NAFLD, the lipid-lowering drug that enhances insulin resistance commonly reduced hepatic steatosis [5]. Also, antioxidants have critical roles in prevention of diseases, but still need in-depth investigations [6].

Stigma of *Crocus sativus* flower, also known as saffron, has been utilised as functional food in prevention of diseases. Biological and pharmacological properties of saffron and its active constituent, and their possible therapeutic uses for a broad range of diseases have been extensively examined [7]. Saffron extract (80 mg/kg body weight) improved atherogenic index (lower LDL/HDL level) and significantly reduced plasma total cholesterol level compared to control [8]. Besides, weak to moderate antinociceptive and anti-inflammatory effects of saffron extract were determined based on the chronic inflammation animal model (Wistar rats) that were induced edema by formalin in the rat's paw, where 0.8 g/kg body weight of saffron aqueous extract was injected to the experimental rats [9].

Safranal and crocin are the main bioactives in saffron. Previous study reported that safranal significantly increased liver antioxidant enzymes (superoxide dismutase and glutathione S-transferase) of male aged Wistar rats (10 and 20 months old) after supplementation of safranal (0.5 μg/g body weight) for a month [10]. Crocin is also one of the medicinal compounds of saffron besides safranal. It has been studied for weight loss [11], inhibited oxidative stress [12, 13] as well as improved insulin resistance and blood glucose level [14–16].

Crocin supplementation (80 mg/kg body weight) promoted weight loss by decreasing the rate of body weight gain as well as reduce body fat, plasma triacylglycerol and total cholesterol levels of male Sprague Dawley that fed with a high-fat diet (HFD) for 12 weeks to induce obesity [7]. These beneficial effects of crocin provide a rationale for its use in individual with NAFLD. Due to saffron extract has medicinal effect against several diseases, therefore, we performed selected biochemical analyses and histopathological assay for determining protective effects of crocin-rich saffron extract and crocin supplementation on NAFLD in HFD-induced obese rats. Plasma levels of aspartate transaminase (AST), alanine transaminase (ALT), alkaline phosphatase (ALP) and albumin (ALB) were also determined to test hepatic function of the HFD fed rats.

## Methods

### Plant materials

Saffron (stigma of *C. sativus* flower) used in this study was from Iranian origin. It was purchased from a local retailer in Mashhad, Iran. The crocin powder was purchased from Sigma-Aldrich (M) Sdn Bhd (Selangor, Malaysia). This plant had been identified by Ms Molaei from Ferdowsi University. The voucher sample was kept in a reference herbarium at the Faculty of Pharmacy, Mashhad University of Medical Sciences, and the voucher specimen number is 134–0319–1.

### Preparation and quantification of crude extract

Preparation and quantification of a crude ethanolic extract of saffron were done according to our previously published method [8]. Presence of crocins including alpha-crocin, crocin 2, crocin 3, crocin 4, crocin 5 and crocin 6 was detected at 440 nm, and safranal was determined at 308 nm in the extract. The saffron extract used in this study contained total crocin of 29 g/100 g DW (dry weight) and safranal of 1.9 g/100 g DW [8]. It was estimated that high dose (80 mg/kg) and low dose (40 mg/kg) of saffron extract supplementation groups received daily 23.2 and 11.6 mg of crocin per kg body weight, respectively.

### Animals and diet

Animal experimental procedures were approved by the Institutional Animal Care and Use Committee of Universiti Putra Malaysia. Study was conducted following the international principles for laboratory animal use and care. A total of 36 healthy male Sprague Dawley rats at 8 weeks old, weighed 200–250 g were used in this survey. Each experimental group consisted six rats, where all the rats were purchased from the Faculty of Veterinary Medicine, Universiti Putra Malaysia. Each rat was housed and acclimatised in a temperature controlled room of 25 °C in individual cage, and on a 12:12-h dark–light cycle. The bedding of each cage was changed every 3 days and all rats were given tap water ad libitum. All experimental rats were fed with normal (5 % fat) and high-fat (40 % fat) diets to induce obesity. Ingredients of the rat diets are shown in Table 1. After obesity induction, the rats were randomly allocated into control and treatment groups as follows:

**Table 1:** Formulations of normal and high-fat diets

Ingredient	Normal diet (g/kg diet)	High-fat diet (g/kg diet)
Corn starch	650	150
Casein	200	200
Beef tallow	0	400
Corn oil	50	0
Sucrose	0	150
Cellulose	50	50
Mineral mix	35	35
Vitamin mix	10	10
DL-Methionine	3	3
Choline bitarate	2	2

*Control groups:*

1. Normal diet (ND)
2. High-fat diet (HFD)

*Treatment groups:*

(3) High-fat diet + crocin 40 mg/kg (HFD + L-CRO)
(4) High-fat diet + crocin 80 mg/kg (HFD + H-CRO)
(5) High-fat diet + saffron extract 40 mg/kg (HFD + L-SAF)
(6) High-fat diet + saffron extract 80 mg/kg (HFD + H-SAF)

Normal and high-fat diets were given to control rats without addition of saffron extract and crocin, whereas treatment groups were fed with specially prepared pellet added with saffron extract or crocin. The saffron extract and crocin of two different doses (40 & 80 mg/kg/day) were supplemented to the rats by homogeneously mixing the extract or crocin to the dough of HFD. The dough was shaped, dried and stored in the dard room before feeding the experimental rats.

### Food intake

Amount of food consumed daily was measured for all control and treatment groups from the quantity of feed supply and the amount remaining by end of each experimental day.

### Blood collection and organ preparation

At the end of experimental period, the rats were fasted overnight (12 h) and then sacrificed after ether anaesthesia. Blood was collected into dry clean centrifuge tubes and plasma was separated by centrifuging at 3000 rpm for 15 min. Plasma samples were kept frozen for biochemical analyses. The rats were thereafter quickly sacrificed and livers were collected, dried on tissue and individually weighed for each rat.

### Relative liver weight

Throughout the experiment, body weight of all experimental rats was recorded weekly. At the end of the experiment, body weight and liver weight of all rats from control and treated groups were measured and recorded. Relative liver weight was calculated using following equation:

$$ \mathrm{Relative}\kern0.28em \mathrm{liver}\kern0.28em \mathrm{weight}=\frac{\mathrm{Absolute}\kern0.28em \mathrm{liver}\kern0.28em \mathrm{weight}\kern0.28em \left(\mathrm{g}\right)}{\mathrm{Body}\kern0.28em \mathrm{weight}\kern0.28em \mathrm{of}\kern0.28em \mathrm{rat}\kern0.28em \mathrm{on}\kern0.28em \mathrm{sacrifice}\kern0.28em \mathrm{day}\kern0.28em \left(\mathrm{g}\right)}\times 100 $$

### Biochemical analysis

After 8 weeks of treatment with saffron extract and crocin, plasma of the experimental rats was further tested for selected biochemical parameters. In this study, hepatic function of the experimental rats was evaluated based on plasma levels of aspartate transaminase (AST), alanine transaminase (ALT), alkaline phosphatase (ALP) and albumin (ALB) which were determined by colorimetric assay using COBAS C 311 Analyzer by Roche Diagnostics (Basel, Switzerland).

### Histopathological analysis

Pieces of tissue samples from right lobe of liver taken from each rat were fixed in 10 % buffered formalin, routinely administered and fixed in paraffin wax. Embedded paraffin sections (5 μm) were then cut and stained with haematoxylin and eosin (H&E). For each rat, five slides were examined using a light microscope. Quantitative assessments of liver samples were done using validated scoring systems for NAFLD [17], where the scoring systems are shown in Table 2.

**Table 2:** Histopathological scoring system for nonalcoholic fatty liver disease (NAFLD) [17]

Component	Grade 0	Grade 1	Grade 2	Grade 3	Range
Steatosis	<5 %	5–33 %	34–66 %	>67 %	0–3
Hepatocyte ballooning	0	Few	Many	N/A	0–2
Lobular inflammation	0	<2 foci/ 20×	2–4	>4	0–3
NAFLD activity score (NAS)	–	–	–	–	0–8

### Statistical analysis

Data were presented as mean ± standard error of the mean (SEM). One-way analysis of variance coupled with Duncan’s multiple range test was used to determine statistical differences between the mean values using SPSS statistical software version 16. *P* values of less than 0.05 were considered statistically significant (*p* < 0.05).

## Results

### Relative liver weight of rats and food intake

Absolute liver weight, as well as relative liver weight of experimental rats, were calculated (Table 3). The relative liver weights between ND and HFD﻿ control groups were significantly different (*p* < 0.01). Saffron extract (80 mg/kg) was found to reduce the liver weight of HFD-induced obese rats. Although the result revealed that liver weight was not adversely affected by crocin treatment, high-dose crocin (80 mg/kg/day) treated group had a slight increment of relative liver weight. Food intakes during 8 weeks treatment of experimental rats are summarised in Table 4.

**Table 3:** Effect of saffron extract and crocin on relative organs weight of rats

Groups	Absolute liver weight	Relative liver weight
ND	9.58 ± 0.71	2.28 ± 0.19
HFD	17.58 ± 1.21	3.07 ± 0.32^# #^
HFD + L-CRO	16.28 ± 3.48	2.75 ± 0.23
HFD + H-CRO	16.46 ± 3.67	3.03 ± 0.23
HFD + L-SAF	15.42 ± 3.64	2.75 ± 0.28
HFD + H-SAF	13.74 ± 2.48	2.46 ± 0.25^**^

Values are expressed as mean ± SEM of six rats

*p < 0.05, **p < 0.01 for negative control (HFD)

^#^ *p* < 0.05, ^# #^ *p* < 0.01 for normal control (ND)

**Table 4:** Effect of the saffron extract and crocin on food intake during 8 weeks of treatment

Week of treatment	Food intake (g)					
	ND	HFD	HFD + L-CRO	HFD + H-CRO	HFD + L-SAF	HFD + H-SAF
0	132.4 ± 2.8	115.6 ± 9.5	106.8 ± 7.8	114.2 ± 4.6	111.3 ± 16.2	114.5 ± 13.7
1	135.1 ± 3.4	106.1 ± 8.2	94.8 ± 12.7	97.6 ± 9.7	101.5 ± 11.7	96.3 ± 14.4
2	135.1 ± 6.1	112.6 ± 4.6	105.6 ± 19.3	105.6 ± 19.3	103.8 ± 13.3	106.6 ± 8.8
3	133.2 ± 3.4	112.4 ± 7.2	105.1 ± 14.4	94.6 ± 5.4	100.3 ± 12.6	99.6 ± 5.5
4	130.1 ± 7.4	110.7 ± 5.5	112.1 ± 12.3	110.8 ± 16.3	109.3 ± 11.7	109.0 ± 9.5
5	132.7 ± 0.9	111.4 ± 6.4	110.7 ± 12.5	100.1 ± 6.6	103.1 ± 14.4	104.4 ± 2.1
6	125.1 ± 11.5	102.1 ± 9.9	98.3 ± 13.4	93.5 ± 7.1	96.6 ± 14.9	87.6 ± 4.7
7	127.6 ± 11.3	109.8 ± 17.9	106.8 ± 4.5	110.1 ± 10.2	100.5 ± 11.5	100.8 ± 5.8
8	132.3 ± 9.1	120.1 ± 3.6	104.7 ± 0.5	103.6 ± 3.5	103.5 ± 6.1	100.5 ± 1.1*

Values are expressed as mean ± SEM of six rats

*p < 0.01 for negative control (HFD)

### Biochemical analysis

**Fig. 1:**
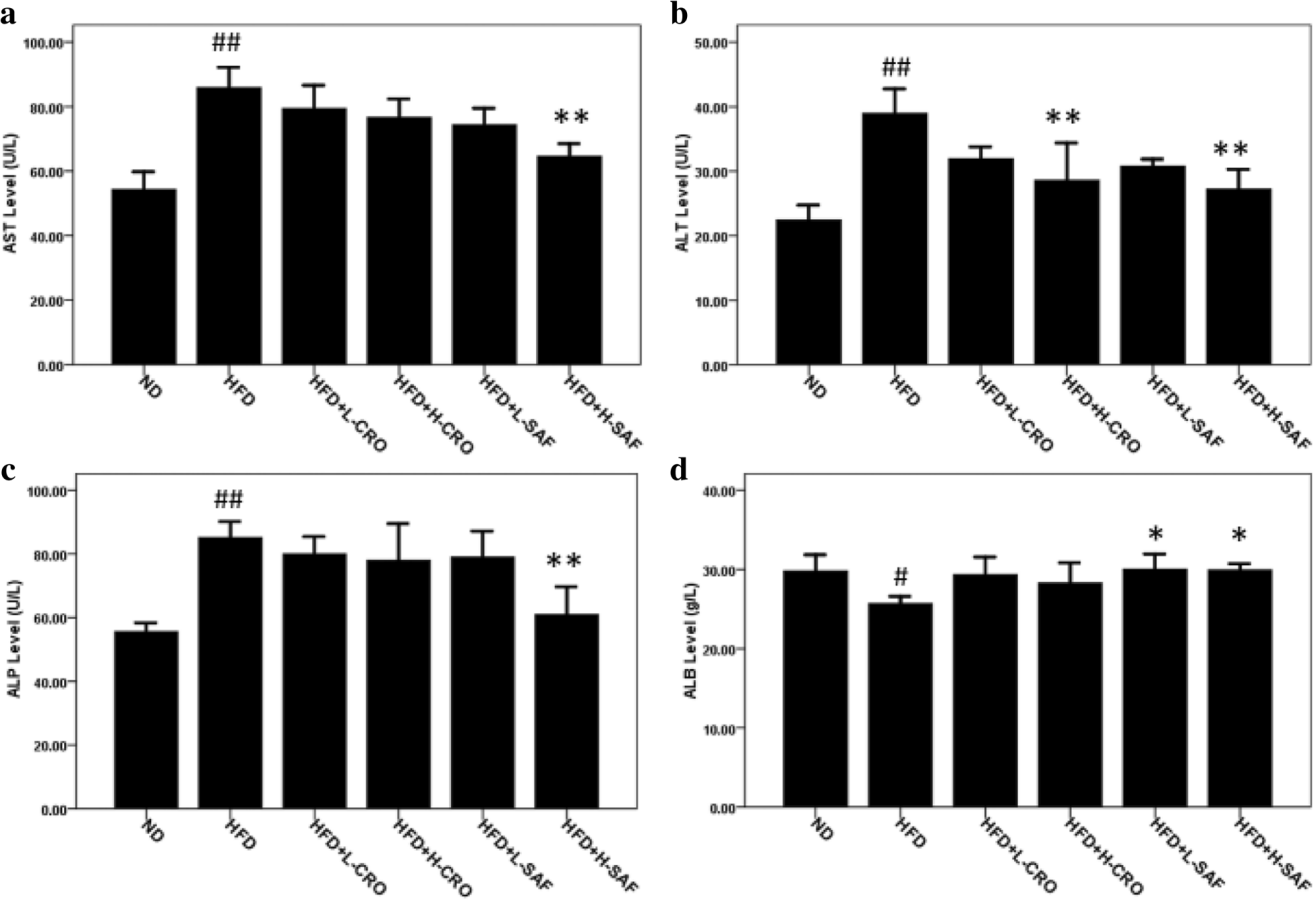
Effect of saffron extract and crocin on plasma biochemical analyses. **a** Aspartate transaminase (AST); **b** alanine transaminase (ALT); **c** alkaline phosphatase (ALP); **d** albumin (ALB). Values are expressed as mean ± SEM (*n =* 6); *p < 0.05 and **p < 0.01 for high-fat diet control (HFD); ^#^ *p* < 0.05 and ^##^ *p* < 0.01 for normal control (ND)

Changes in liver enzymes of obese male rats supplemented with saffron extract and crocin at low and high doses (40 and 80 mg/kg) are indicated in Fig. 1. Results show that there were significantly changed for AST, ALT and ALP (*p* > 0.01), as well as ALB (*p* > 0.05) between normal and HFD control groups. Oral administration of saffron extract at a high concentration (80 mg/kg) for 8 weeks showed significant reductions in ALT, AST and ALP levels, whereas crocin (80 mg/kg) group had a significant decrease in ALT compared to HFD control group. Moreover, saffron extract significantly improved level of ALB of the obese rats compared to HFD control rats (*p* > 0.05) (Fig. 1).

### Histopathological analyses

**Fig. 2:**
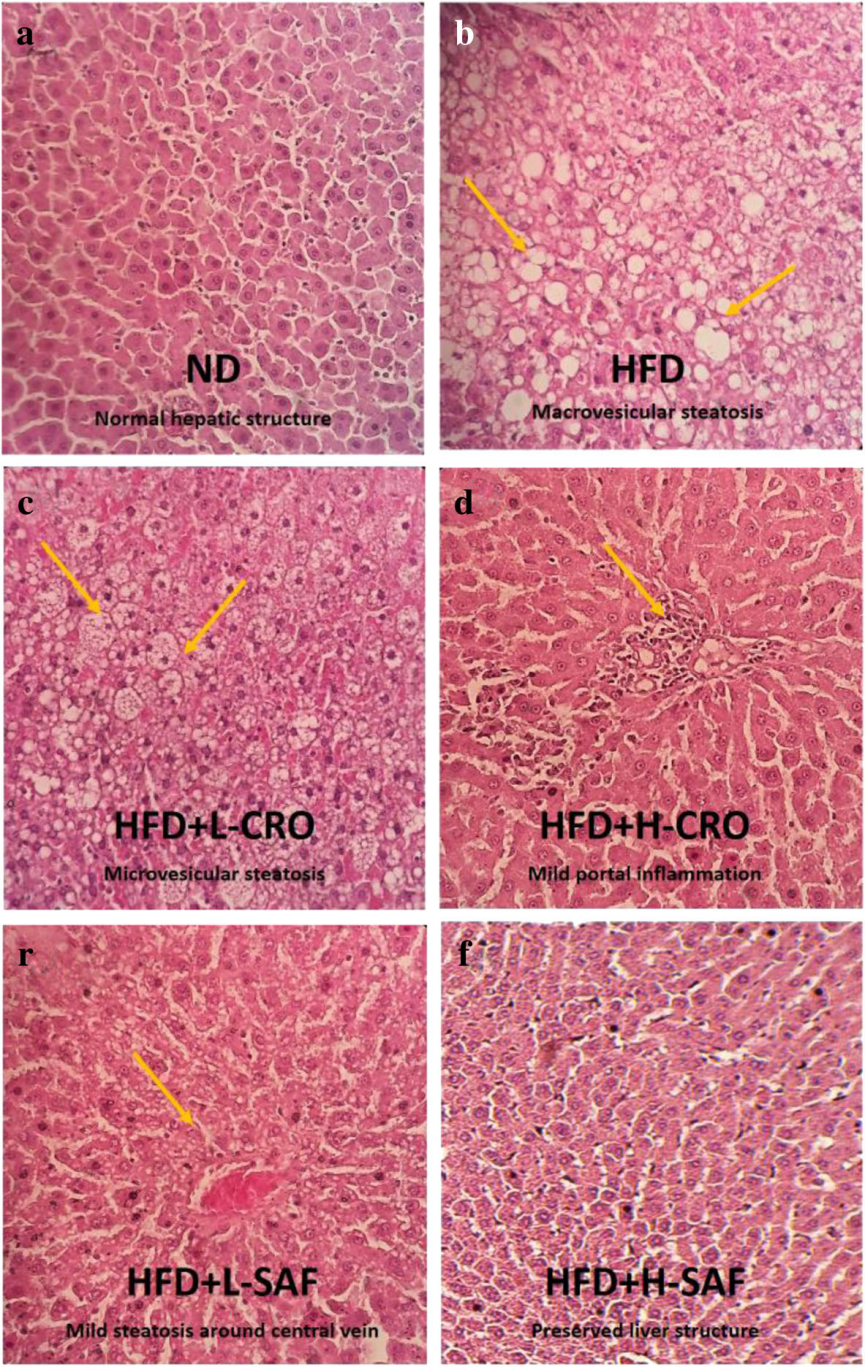
Effect of saffron extract and crocin on liver steatosis based on histopathological examination (H&E staining). Representative histopathological examination of H&E staining of liver tissue prepared from experimental rats fed with **a** normal diet (ND), **b** high-fat diet (HFD), **c** high-fat diet + crocin 40 mg/kg (HFD + L-CRO), **d** high-fat diet + crocin 80 mg/kg (HFD + H-CRO), **e** high-fat diet + saffron extract 40 mg/kg (HFD+ L-SAF), and **f** high-fat diet + saffron extract 80 mg/kg (HFD + H-SAF) (magnification 400×). Major histopathological changes induced by HFD in rat liver were hepatosteatosis, ballooning and inflammation of hepatocytes

Histopathological examination of NAFLD was typically presented by steatosis, hepatocyte ballooning, portal and lobular inflammation. Micrographs in Fig. 2a, f show normal hepatic structure and micrographs in Fig. 2b-e reveal the fibrosis and steatosis of hepatocytes of obese rats fed with HDF.

As shown in Fig. 2, micrograph (b) shows severe hepatosteatosis condition of the experimental rats fed with HDF, where many hepatocytes in acinar zone III had ballooning and a mix inflammatory cell infiltration; micrograph (c) reveals a mild microvascular steatosis of liver tissues of the obese rats treated with crocin, where lobular inflammation and hepatocellular ballooning can be observed, whereas micrograph (d) shows a severe fibrosis of hepatocytes with mild steatosis. Besides, micrograph (e) shows a mild fibrosis around central vein with no steatosis observed.

Interestingly, crocin and saffron extract supplementations were dose-dependently reduced hepatic steatosis with minor ballooning and scattered inflammation (Fig. 2c-f). Quantitative assessment of fatty liver tissues of the obese rats that supplemented with saffron extract and crocin indicated the hepatic steatosis and ballooning were significantly improved, especially the high dose supplementation of saffron extract (*p* < 0.01) and crocin (*p* < 0.05). In term of NAFLD activity score (NAS), saffron extract had dose-dependently improved NAS values, and 80 mg/kg of crocin ameliorated the scores (Table 5).

**Table 5:** Quantitative histopathological assessment of fatty liver tissues for rats fed with saffron extract and crocin

Groups	Steatosis	Ballooning	Inflammation	NAS
ND	0	0	0.33 ± 0.51	0.33 ± 0.51
HFD	2.66 ± 0.51^# #^	1.66 ± 0.51^# #^	1.50 ± 1.04	5.83 ± 1.47^# #^
HFD + L-CRO	1.66 ± 0.81	1.33 ± 0.51	1.00 ± 0.63	4.00 ± 1.41
HFD + H-CRO	1.33 ± 1.03*	0.66 ± 0.51*	1.33 ± 1.03	3.16 ± 1.32*
HFD + L-SAF	1.50 ± 0.54	0.50 ± 0.54**	0.66 ± 0.51	2.66 ± 0.81**
HFD + H-SAF	0.83 ± 0.75**	0.16 ± 0.40**	0.50 ± 0.54	1.50 ± 1.22**

For each scoring slide, a five-field randomly selection was considered

Scores are expressed as mean ± SEM of six rats

*p < 0.05, **p < 0.01 for negative control (HFD)

^#^ *p* < 0.05, ^# #^ *p* < 0.01 for normal control (ND)

## Discussion

Similar to numerous human diseases, fatty liver in rodents is diet-inducible [18]. ﻿HFD increases body weight and causes diabetes in different strains of rodent [8, 19]. HFD can also increase level of liver fat and hepatic insulin resistance more rapid than increment in peripheral fat deposition [20]. ﻿﻿Development of fatty liver induced by HFD is associated with increases in the levels of serum AST and ALT [9, 21].

In this study, after implementation of obesity induction phase among experimental rats, we evaluated hepatic implications of crocin and ethanolic extract of saffron at doses of 40 and 80 mg/kg body weight that orally administered to HFD induced obese rats based on a daily basis for 56 days (8 weeks). Increased liver weight (Table 3), highly elevated levels of AST and ALT (Fig. 1a-b) and the observation obtained from microscopic examination of liver tissue indicated that HFD caused hepatic steatosis and injury to rats’ liver.

Result from biochemical evaluation shows that supplementations of saffron extract and crocin were dose-dependently reduced plasma ALT and AST levels of the HFD-fed rats. It shows that saffron extract together with crocin exerts protection against hepatic damage in HFD-induced obese rats. ﻿﻿A high level of plasma ALP is typically found in the animals with cholestatic liver disease and also induced by hepatotoxic agents [22]. The significant reduction in plasma ALP level of the saffron extract (80 mg/kg) supplemented rats supports the non-occurrence of cholestasis to experimental rats at the extract dose tested.

Histopathological findings of the liver samples demonstrated protective effect of saffron extract at concentration of 80 mg/kg body weight against NAFLD. The hepatoprotective activity of saffron against fatty liver could be due to modulation of liver enzymes in parallel with major normalisation of liver size and structure as well as a distinct reduction of fatty infiltration in hepatocytes of the HFD induced obese rats.

Although this study is the first time evaluation of protective effect of saffron extract and its most bioactive compound, crocin, among experimental rats with diet-induced fatty liver, however, the relevant studies [23–26] support the findings of this study that saffron is a potential nutraceutical for protecting liver tissue from hepatic steatosis.

## Conclusion

Saffron extract contains crocin as the main bioactive compound. Overall biochemical and histopathological outcomes suggest that saffron extract and crocin supplementations at the tested concentrations maintained liver function and alleviated hepatosteatosis in HFD induced obese rats, which are encouraging. A more definitive evidence of the protective effects of saffron and crocin is needed before saffron can generally be recommended for treatment of fatty liver disease.

## Abbreviations

ALB: Albumin
ALP: Alkaline phosphatase
ALT: Alanine transaminase
AST: Aspartate transaminase
CHD: Coronary heart disease
DW: Dry weight
H&E: Haematoxylin and eosin
HDL: High-density lipoprotein
HFD: High-fat diet
HFD + H-CRO: High-fat diet + crocin 80 mg/kg
HFD + H-SAF: High-fat diet + saffron extract 80 mg/kg
HFD + L-CRO: High-fat diet + crocin 40 mg/kg
HFD + L-SAF: High-fat diet + saffron extract 40 mg/kg
LDL: Low-density lipoprotein
NAFLD: Nonalcoholic fatty liver disease
NAS: NAFLD activity score
ND: Normal diet
T2DM: type 2 diabetes mellitus
